# Hypoxic Three-Dimensional Scaffold-Free Aggregate Cultivation of Mesenchymal Stem Cells in a Stirred Tank Reactor

**DOI:** 10.3390/bioengineering4020047

**Published:** 2017-05-23

**Authors:** Dominik Egger, Ivo Schwedhelm, Jan Hansmann, Cornelia Kasper

**Affiliations:** 1 Department of Biotechnology, University of Natural Resources and Life Sciences, Muthgasse 18, 1190 Vienna, Austria; dominik.egger@boku.ac.at; 2 Translational Center, University Hospital Wuerzburg, Roentgenring 11, 97070 Wuerzburg, Germany; ivo.schwedhelm@uni-wuerzburg.de (I.S.); jan.hansmann@uni-wuerzburg.de (J.H.)

**Keywords:** mesenchymal stem cells, scaffold-free, aggregate cultivation, stirred tank reactor, dynamic cultivation, hypoxia, stemness, computational fluid dynamics

## Abstract

Extensive expansion of mesenchymal stem cells (MSCs) for cell-based therapies remains challenging since long-term cultivation and excessive passaging in two-dimensional conditions result in a loss of essential stem cell properties. Indeed, low survival rate of cells, alteration of surface marker profiles, and reduced differentiation capacity are observed after in vitro expansion and reduce therapeutic success in clinical studies. Remarkably, cultivation of MSCs in three-dimensional aggregates preserve stem cell properties. Hence, the large scale formation and cultivation of MSC aggregates is highly desirable. Besides other effects, MSCs cultivated under hypoxic conditions are known to display increased proliferation and genetic stability. Therefore, in this study we demonstrate cultivation of adipose derived human MSC aggregates in a stirred tank reactor under hypoxic conditions. Although aggregates were exposed to comparatively high average shear stress of 0.2 Pa as estimated by computational fluid dynamics, MSCs displayed a viability of 78–86% and maintained their surface marker profile and differentiation potential after cultivation. We postulate that cultivation of 3D MSC aggregates in stirred tank reactors is valuable for large-scale production of MSCs or their secreted compounds after further optimization of cultivation parameters.

## 1. Introduction

In the context of regenerative medicine, mesenchymal stem cells (MSCs) are still considered the most promising and eligible candidate for therapeutic use in cell-based therapies. Their regenerative potential is based on high proliferative activity, the capacity to differentiate into specific cell types of the musculoskeletal and connective tissue [1,2] as well their ability to specifically migrate to injured tissue sites, where they are involved in tissue repair and anti-inflammatory effects [3] via delivery of trophic factors [4,5,6]. Consequently, this results in immunosuppressive effects, enhanced tissue repair, and angiogenesis. Therefore, it is essential to maintain their inherent properties during ex vivo cultivation to enable for therapeutic success and reproducibility in clinical studies. 

However, altered immune properties and low in vivo survival rates of MSCs were reported after ex vivo expansion [7,8]. Still, large-scale expansion of MSCs is usually carried out in two-dimensional (2D) static conditions, which was shown to alter their inherent immunophenotype [9]. In contrast, the formation of three-dimensional (3D) MSC aggregates seems to preserve their phenotype and differentiation potential [10,11]. Furthermore, increased secretion of proangiogenic factors and anti-inflammatory cytokines was observed after aggregate formation [12,13]. Therefore, the upscale of aggregate formation and cultivation for the therapeutic use of cells or their secreted compounds is desirable. The cultivation of aggregates in small-scale systems like microtiter plates or hanging drops is well established [14]. However, aggregates display nutrient and oxygen gradients from surface to core which was demonstrated to result in a necrotic core for aggregates >500 µm [15]. Therefore, dynamic cultivation systems that enable enhanced mass transfer seem preferable for aggregate cultivation. Indeed, cultivation of MSC aggregates in rotating wall vessel bioreactors, shake flasks, spinner flasks, or on an orbital shaker did not result in necrotic tissue [11,16,17,18]. However, no study reports the cultivation in a stirred tank reactor.

Since the natural in vivo environment of MSC often displays oxygen concentrations considerably <21% O_2_ (hypoxia) [19,20,21], the effect of reduced oxygen conditions on MSCs was extensively investigated in the past and regarding the therapeutic use of MSCs several advantages of hypoxic cultivation emerged [22]. In fact, MSCs exhibit increased proliferation [21,23,24], reduced senescence [25], and prolonged genetic stability [26], when exposed to hypoxia while maintaining their immunosuppressive properties [27]. 

Therefore, in the present study, we cultivated human adipose derived 3D MSCs aggregates in a continuously stirred tank reactor (CSTR) under normoxic (21% O_2_) and hypoxic (5% O_2_) conditions. Since rather high shear forces can occur in a stirred tank reactor, the actual shear stress was estimated via computational fluid dynamics. After cultivation, surface marker expression and differentiation capacity was evaluated. 

Although average shear forces of 0.2 Pa were present, MSCs did not differentiate spontaneously and maintained their innate phenotype and trilineage differentiation potential. Cultivation of aggregates in a CSTR might be beneficial for large scale expansion of MSCs, production of MSC aggregates, or their secreted trophic factors. To our knowledge, this is the first study that demonstrates the formation and cultivation of MSC aggregates in a CSTR.

## 2. Materials and Methods

### 2.1. Bioreactor Design

For the cultivation of human MSCs, a continuously stirred tank reactor (CSTR) was created by computer-aided design (CAD) software (Solidworks, Dassault Systèmes, Stuttgart, Germany). Based on the CAD drawings, all parts of the reactor framework were manufactured from stainless steel. As an exception, the impeller (impeller diameter *d* = 25 mm) was constructed from polyether ether ketone (PEEK) (GT Labortechnik, Arnstein, Germany). The custom-made round-bottom glass vessel (vessel diameter *D* = 80 mm, round bottom radius *r* = 40 mm) and riser pipes were hand manufactured by a local glassblower (Glaspunkt, Burghausen, Germany). The reactor bottom clearance was set equally to the impeller diameter *d*. In order to minimize the risk of contamination of the vessel interior, the stirring shaft was further equipped with a mechanical seal (Trelleborg Sealing Solutions, Stuttgart, Germany). For measuring the oxygen content in the culture medium during hypoxic and normoxic ambient conditions, an optical oxygen sensor spot was attached to the reactor glass wall (Presens GmbH, Regensburg, Germany).

### 2.2. Compuitational Fluid Dyamics

The bioreactor CAD schematics were harnessed to establish a computational fluid dynamics (CFD) model from which data on flow field and shearing were collected. The CAD model files were imported into a suitable finite element method (FEM) software (Comsol Multiphysics 5.2, Comsol Multiphysics GmbH, Göttingen, Germany) and processed for subsequent FEM computations. Briefly, the material properties of the model geometry were adjusted to comply with human MSC culture medium at 37 °C (dynamic viscosity 0.765 mPa·s, density 998 kg/m³).

For flow velocity field computations, the Comsol rotating machinery module was used to solve the resulting set of Navier–Stokes differential equations. First, the rotating model domain was defined as such by parameterizing its rotation velocity. Here, the κ–ε turbulence model was assumed and the corresponding model constants for turbulence fluid flow were set to *C_ε1_* = 1.44, *C_ε2_* = 1.92, *C_μ_* = 0.09, *σ_κ_* = 1.0, *σ_ε_* = 1.3, Von Kármán constant *K_N_* = 0.41, and wall roughness *B* = 5.2. A pressure point constraint was defined at the model surface boundary to set ambient pressure conditions. Furthermore, a symmetry boundary condition was applied to the fluid–gas interface. Wall functions were defined for all remaining boundaries. An auxiliary sweep was performed in order to stabilize solver convergence. Therefore, the dynamic viscosity of the fluid was subsequently multiplied by a numerical auxiliary factor defined as *visc_fac.* For the first computation iteration, a value of *visc_fac* = 50 was set. Following, computations for the steady-state solution were performed while stepwise lowering the auxiliary factor down to a value of *visc_fac* = 1.

### 2.3. Cell Culture

The use of human tissue was approved by the ethics committee of the Medical University Vienna, Austria (EK Nr. 957/2011, 30 January 2013) and the donor gave written consent. Human ASCs were isolated within 3 h after surgery as described before from a female donor [21] (48 years old). Briefly, fat tissue was minced with scissors and digested with collagenase type I (Sigma Aldrich, St. Louis, MO, USA). Subsequently, multiple centrifugation and washing steps were carried out to receive the stromal vascular fraction which was then transferred to cell culture flasks. ASCs were cultivated in standard medium composed of MEM alpha (Thermo Fisher Scientific, Waltham, MA, USA), 0.5% gentamycin (Lonza, Basel, Switzerland), 2.5% human platelet lysate (PL BioScience, Aachen, Germany) and 1 U/ml heparin (Ratiopharm, Ulm, Germany) in a humidified incubator at 37 °C, 5% CO_2_ and 21% (normoxic) or 5% O_2_ (hypoxic). For cryo-preservation, cells were detached by accutase treatment (GE healthcare, Little Chalfont, UK) and transferred to cryo-medium composed of 77.5% αMEM, 12.5% HPL, 10% DMSO (Sigma Aldrich), and 1 U/ml heparin as described before [9] for storage in liquid nitrogen. For bioreactor cultivation, cells were thawed, expanded until passage 2, and harvested by accutase treatment. Cells for cultivation at 5% O_2_ have also been isolated and subcultivated at 5% O_2_ until seeding.

### 2.4. Bioreactor Cultivation

After steam sterilization, the CSTR was filled with PBS at 37 °C in order to calibrate the PreSens oxygen sensor. The tank was filled and emptied through one of the ports in the lid while the lid itself was kept closed at all time. After calibration, PBS was removed with a suction pump and the tank was filled with 130 mL of a 1 × 10^5^ cells/mL single cell suspension of MSCs (13 × 10^6^ cells total) in standard medium with 10 instead of 2.5% human platelet lysate (PL Bioscience, Aachen, Germany) was used. Cells were cultivated for six days at 600 revolutions per minute (rpm), 37 °C, 5% CO_2_, and 21% or 5% O_2_. After three days, 100 mL of the medium was replaced. Aggregates and cells were allowed to sediment for 15 min prior to medium change. 

After six days, the medium containing cells and aggregates was transferred to 50 mL centrifugation tubes, the tank was rinsed with 40 mL PBS, the PBS added to the tubes and the tubes centrifuged for 5 min at 500× *g*. The bioreactor tank was filled with 25 mL of a 37 °C pre-warmed Accumax solution (Sigma Aldrich, St. Louis, MS, USA) and incubated 15 min at 37 °C in order to remove adherent cells from the glass wall. In parallel, supernatant from centrifugation tubes was removed, the pellets resuspended in 40 mL PBS, unified in one tube, and again centrifuged for 5 min at 500× *g*. After removal of the supernatant, the pellet was resuspended in Accumax solution from the bioreactor tank. After this, the solution was incubated 15 min in a 37 °C water bath and further for 30 min on a horizontal shaker at 300 rpm and 37 °C in order to dissociate the aggregates. Then, the cell suspension was passed through a cell strainer to separate single cells from remaining aggregates. Cells were counted by trypan blue staining with a hemocytometer after incubation to determine cell number and viability (overall cells were incubated for 45 min in Accumax solution). Furthermore, the cell strainer was placed in a 6-well plate and incubated in 6 mL Accumax solution for 1 h at 100 rpm and 37 °C. Single cells released from the aggregates were also counted by trypan blue staining. Harvested ASCs were frozen as described above for further analysis of surface markers and differentiation capacity.

### 2.5. Phenotyping

To determine MSC surface marker expression cells were detached by accutase treatment and stained with MSC phenotyping kit (Miltenyi Biotech GmbH, Bergisch Gladbach, Germany) according to manufacturer’s instructions. Stained cells (5 × 10^5^ cells per aliquot) were resuspended in 300 µL flow cytometry buffer and acquisition was carried out on a Gallios flow cytometer (Beckman Coulter, Brea, CA, USA). Between 1–5 × 10^4^ gated events were recorded. Subsequent analysis was performed with Kaluza Flow Cytometry software (version 1.3, Beckman Coulter, Brea, CA, USA). 

### 2.6. Differentiation

To evaluate the differentiation capacity of ASCs after cultivation in the CSTR, cells were thawed and cultivated in cell culture flasks to approximately 80% confluency. Subsequently, cells were detached by accutase treatment and seeded into fibronectin coated 12-well plates (BD Bioscience, Franklin Lakes, NJ, USA) at a density of 4000 c/cm^2^. When cells reached confluency, the medium was changed to adipogenic, chondrogenic (both Miltenyi Biotec GmbH, Bergisch Gladbach, Germany), or osteogenic medium (standard medium supplemented with 5 mM beta-glycerolphosphate, 0.1 μM Dexamethasone, 200 µM L-ascorbate-2-phosphate, all from Sigma Aldrich, St. Louis, USA) respectively. Cells were cultivated for 21 days and medium was changed every 2–3 days. Afterwards, cells from chondrogenic and osteogenic differentiation were fixated with 96% ethanol while cells from adipogenic differentiation were fixated with 4% paraformaldehyde for further histological staining. 

### 2.7. Histologic Stainings

ASCs cultivated in adipogenic medium were stained with Oil Red O (staining of lipid vacuoles). For this, cells were rinsed with ddH_2_O and incubated in Oil Red O solution (Sigma Aldrich) for 20 min. ASCs cultivated in chondrogenic medium were stained with alcian blue (staining of glycosaminoglycans). Briefly, cells were rinsed with 3% acetic acid and incubated in alcian blue solution (1% *w/v* alcian blue in 3% acetic acid) for 30 min. ASCs cultivated in osteogenic medium were double stained with DAPI (Sigma Aldrich) and calcein (staining for calcium, Franklin) and silver nitrate (also known as von Kossa; staining for phosphates). For fluorescent double staining, cells were rinsed with PBS and incubated in DAPI solution (4 µL/mL DAPI in PBS). Subsequently, cells were rinsed with ddH_2_O and incubated in calcein solution (5 µg/mL) over night at 4 °C. Cells for staining of phosphates were rinsed with ddH_2_O and incubated in 5% silver nitrate solution (Carl Roth) for 30 min in the dark. Subsequently, cells were rinsed again, exposed to UV light for 2 min, and rinsed with decolorization solution (5% Na_2_CO_3_, 0.2% formaldehyde in ddH_2_O). 

### 2.8. Statistical Analysis

All results are presented as mean ± standard deviation (SD). Comparisons were carried out by the unpaired, two-sided *t*-test. Values of *p* < 0.1 with a confidence interval of 90% were defined as statistically significant (as indicated by an asterisk). All analysis were carried out with GraphPad Prism 6.01 (GraphPad Software, Inc., La Jolla, CA, USA).

## 3. Results

### 3.1. Shear Stress Estimation By Computational Fluid Dynamics

**Figure 1:**
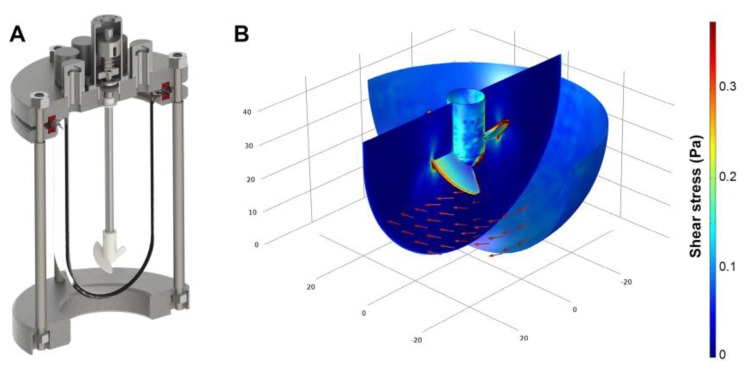
(**A**) Three-dimensional model of the stirred tank reactor used for aggregate cultivation; (**B**) Flow field direction (red arrows) and shear stress distribution (color legend) at a rotational speed of 600 rpm as estimated by computational fluid dynamics.

The impeller of the continuously stirred bioreactor was designed to provide gentle, yet thorough mixing at low shear when operated at moderate impeller speed in the range of 100–120 rpm. However, in order to obviate cells from attaching to the bioreactor glass wall, vigorous stirring rates were necessary. The increase of the impeller rotation frequency to 600 rpm leads to considerable magnitudes of shear stress. To evaluate the impact of high rotational velocities, computational fluid dynamics were used to compute the fluid flow regime of the continuously stirred bioreactor. Here, as shown by the color legend in Figure 1, the calculated shear stress is predominantly found in the range between 0.05 Pa and 0.35 Pa. A closer investigation of the computational fluid dynamics results revealed peak stress levels of 2.5 Pa at the impeller blade tips. When considering the entire spatial dimension of the bioreactor, 0.02 Pa were obtained as average shear stress in total.

### 3.2. Bioreactor Cultivation

**Figure 2:**
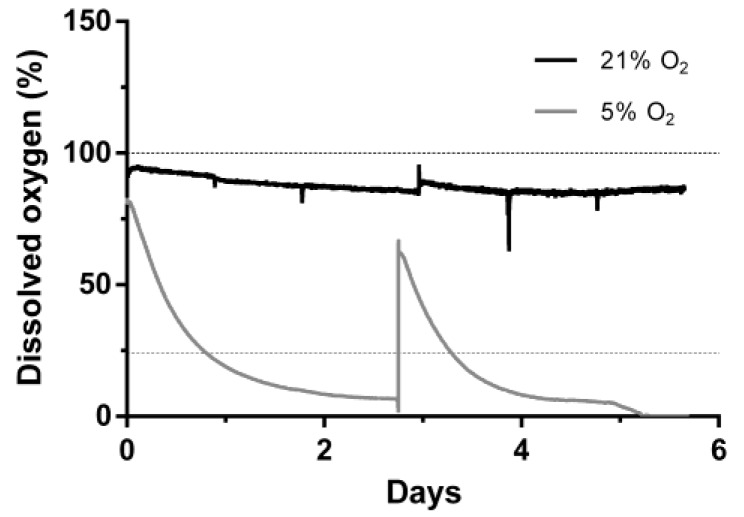
Dissolved oxygen during cultivation mesenchymal stem cells in continuously stirred tank reactor at 21% or 5% O_2_ ambient oxygen. Dashed lines indicate the respective dissolved oxygen concentration at respective equilibrium.

**Figure 3:**
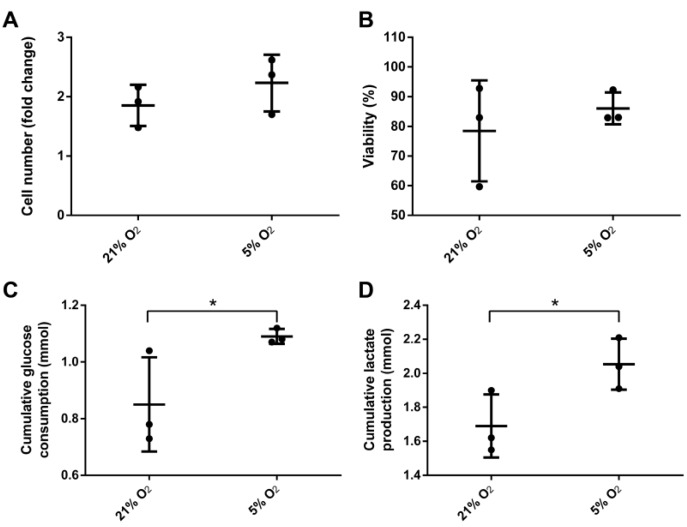
(**A**) Yield of viable cells; (**B**) overall viability, cumulative (**C**) glucose consumption and (**D**) lactate production of mesenchymal stem cells after six days cultivation under 21% and 5% O_2_ in a continuously stirred tank reactor (*n* = 3). Data is represented as mean ± SD, asterisks indicate statistically significant difference (*p* < 0.1, confidence interval of 90%).

Human ASCs were cultivated for 6 days at 21% or 5% O_2_ in a continuously stirred tank reactor. Visible aggregates formed spontaneously after approximately three days. Cells and aggregates were also found to adhere and grow on the glass surface of the bioreactor vessel and partially on the impeller. Dissolved oxygen (DO) decreased slowly under normoxic conditions to approximately 85% until day 6 whereas under hypoxic conditions it decreased to 0% after five days (Figure 2). Cells expanded 1.85-fold (±0.19) under normoxic conditions and 2.23-fold (±0.27) under hypoxic conditions displaying a viability of 78.5 ± 9.8% and 86 ± 3.1% respectively. Human MSCs of different origin displayed an approximately 1.3-fold increased growth rate when cultivated under hypoxic conditions [28]. Although not statistically significant, data of the present study indicate a similar behavior when ASCs are cultivated in a CSTR. Furthermore, glucose consumption (0.85 ± 0.1 mmol) and lactate production (1.69 ± 0.11 mmol) were significantly lower in normoxic conditions compared to hypoxic conditions, where glucose consumption was 1.09 ± 0.02 mmol and lactate production 2.05 ± 0.09 mmol. In the absence of oxygen, glycolytic activity increases since glucose is metabolized rather by lactate acid fermentation than by oxidative phosphorylation in the mitochondria, which reduces the efficiency of ATP production. However, under hypoxic cultivation, cell numbers were slightly increased together with a higher viability (Figure 3).

### 3.3. Stem Cell Properties

**Figure 4:**
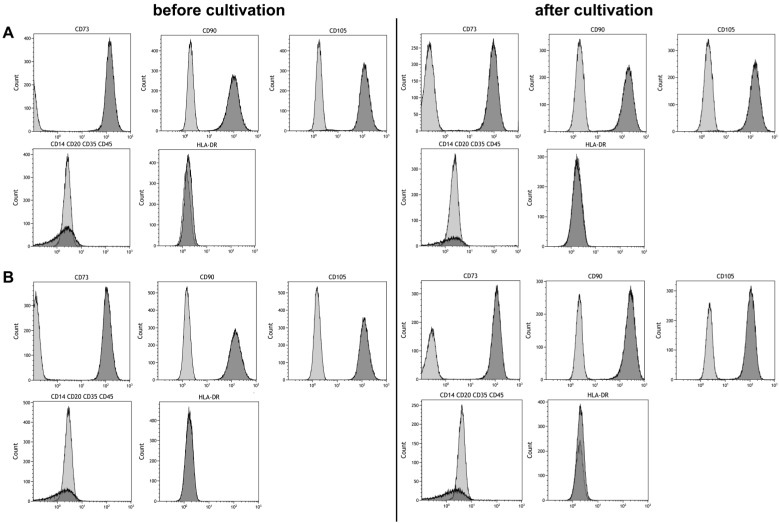
Phenotyping of mesenchymal stem cells before and after six days of cultivation under (**A**) 21% and (**B**) 5% O_2_ in a continuously stirred tank reactor. Light gray areas indicate the isotype control, dark grey areas indicate the phenotype.

**Figure 5:**
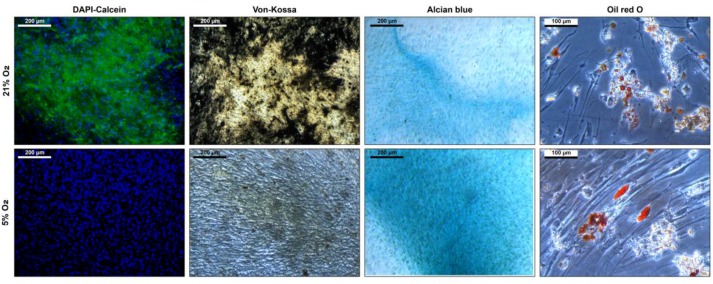
Differentiation of mesenchymal stem cells after six days cultivation under 21% and 5% O_2_ in a continuously stirred tank reactor. Osteogenic differentiation is indicated by DAPI-calcein staining for nuclei and extracellular calcium and von Kossa stain for extracellular phosphates. Chondrogenic differentiation is indicated by alcian blue staining which stains for glycosaminoglycans. Adipogenic differentiation is indicated by Oil Red O staining which stains the intracellular fatty vacuoles.

Maintaining stem cell properties during ex vivo cultivation is mandatory in the context of stem cell expansion for later use in cell-based therapies. Stem cell properties were evaluated by antibody staining of characteristic surface markers that meet the minimal criteria of MSC and evaluation of the differentiation capacity [2]. Surface markers of MSCs before and after cultivation in the CSTR were comparable (Figure 4). Also, surface markers of cells cultivated under normoxic and hypoxic conditions were comparable and met the minimal criteria of MSCs. Furthermore, differentiation into adipogenic, chondrogenic, and osteogenic lineage was observed (Figure 5). However, slightly elevated adipogenic and chondrogenic but a reduced osteogenic differentiation was observed in hypoxic conditions compared to normoxic in the present study. 

## 4. Discussion

Cultivation of MSCs aggregates or spheroids is well-established at the scale of hanging drops [14], microtiter plates [29], or shaking flasks [17,30]. The maintenance of stem cell properties after cultivation on an orbital shaker was already demonstrated in a study with murine MSC aggregates [18]. Another study reported the cultivation of human MSCs in shaking flasks [17]. However, to the best of our knowledge, no reports have been made regarding the upscale of MSC aggregate cultivation in a stirred tank reactor. Therefore, in this study, we investigated the cultivation of MSC aggregates in a CSTR reactor under normoxic (21% O_2_) and hypoxic (5% O_2_) conditions. Cells cultivated under hypoxic conditions displayed increased proliferation, viability (not significant), and glycolytic activity (significant) compared to normoxic conditions. Furthermore, MSCs maintained their stem cell properties as indicated by their immunophenotype (positive for CD73, CD90, CD105 and negative for CD14, CD20, CD35, CD45, and HLA-DR) and multilineage differentiation capacity. To avoid cell adhesion on the glass wall and impeller of the bioreactor the impeller speed was set to 600 rpm. The resulting average shear stress, as estimated by computational fluid dynamics, was found to be 0.02 Pa with peak shear stress of 2.5 Pa. MSCs are known to react to mechanical cues such as fluid shear forces and several studies reported differentiation, when cells were exposed to shear stress as low as 7.6 × 10^−5^ [31] or 0.01 Pa [32]. However, flow cytometry analysis of surface marker expression revealed exclusively undifferentiated stem cells as no side populations were found. Cellular aggregates were shown to be temporally and spatially heterogeneous with regards to cellular architecture and expression of matrix proteins [11,30,33]. Therefore, cells on the aggregate surface might shield the inner cell mass from fluid shearing. Furthermore, cells maintained their trilineage differentiation potential and displayed slightly elevated adipogenic and chondrogenic but a reduced osteogenic differentiation. The majority of studies reported attenuated osteogenic [34] and adipogenic [28], but elevated chondrogenic [26], differentiation under hypoxic conditions although elevated adipogenic differentiation was also observed [35]. 

The traditional approach for the expansion of MSCs is the cultivation on two-dimensional plastic surfaces. Although this approach is simple and reproducible it is time and material consuming to achieve high cell numbers. Also, a rather high surface is required which often results in numerous cell culture vessels and extensive passaging of cells. Due to the comparably high number of opening events the risk of contamination is increased. Therefore, other approaches like the cultivation in hollow-fiber bioreactors with a high surface-to-volume ratio or microcarrier-based systems were developed. For example the quantum system by Terumo expanded 6.6 × 10^8^ MSCs from a 25 mL bone marrow aspirate [36]. Also, expansion of MSCs on microcarrier was performed successfully in spinner flasks [37] and CSTRs [38]. However, the quantum system was mainly developed for use at a clinical scale, and thus might be oversized for research scale while cell harvest is challenging in microcarrier-based systems [39,40]. Also, both approaches are limited by their surface capacity. Though the yield of the presented scaffold-free aggregate cultivation of MSCs is comparably low this system offers a straight forward approach to cultivation of MSCs. Potentially, advantages of traditional suspension culture, such as simplicity, standardizability, rapid development of cultivation protocols, and upscale are translatable to aggregate cultivation. Nevertheless, since homogeneity of aggregates is not given until now, the system may be of interest rather for the production of secreted compounds than cell expansion. Especially, aggregate cultivation under hypoxic conditions might be of benefit in this context since the hypoxic environment in the core of aggregates was reported to increase expression of trophic factors such as VEGF, FGF-2, HGF, and CXCR4 [16,41] as well as ECM proteins such as fibronectin, collagen I, vitronectin, and collagen IV [42]. However, the expression profile of MSC aggregates cultivated in an extensively stirred environment needs further investigation.

In order to improve the cultivation process with regards to yield, viability, and reproducibility further work will focus on the optimization of the bioreactor and impeller geometry. Furthermore, coating agents like silicon will be considered to prevent cell loss due to glass adherence. This might also reduce the required impeller speed and thus contribute to proliferation and viability of cells. Cultivation under hypoxic conditions seems to improve cell yield and viability. However, an oxygen concentration of ≤12% DO (≈2.5% ambient O_2_) should be avoided as it was shown to decrease proliferation of MSCs [21]. Also, serum-free medium was optimized for MSC aggregate cultivation and shown to increase proliferation and similar or enhanced differentiation capacity compared to cultivation in serum-containing medium [18]. 

## 5. Conclusions

We demonstrated the cultivation of 3D MSC aggregates in a CSTR under normoxic and hypoxic conditions. The cultivation under hypoxic conditions resulted in slightly higher yield and viability of cells. Although the exerted shear forces were comparatively high, MSCs maintained their immunophenotype and differentiation capacity. We hypothesize cultivation of MSC aggregates in a CSTR is a viable option for expansion of MSCs or production of secreted compounds after further optimization of cultivation conditions.
